# The flavonoid-sensing regulator AefR is involved in modulating quorum sensing through repressing the MexEF-OprN efflux pump in *Pseudomonas fluorescens*

**DOI:** 10.1128/msystems.00915-24

**Published:** 2025-02-27

**Authors:** Yu Xiao-Quan, Han Jian-Ting, Feng Han-Zhong, Hou Jun, Wang Zhi-Ping, Yong-Xing He

**Affiliations:** 1 Institute of Urology, Gansu Province Clinical Research Center for Urinary System Disease, The Second Hospital & Clinical Medical School, Lanzhou University, Lanzhou, Gansu, China; 2 Ministry of Education Key Laboratory of Cell Activities and Stress Adaptations, School of Life Sciences, Lanzhou University, Lanzhou, Gansu, China; Chinese Academy of Sciences, Beijing, China

**Keywords:** quorum-sensing system, TetR, flavonoids, efflux pumps

## Abstract

Flavonoids, a major component of plant root exudates, play a crucial role in mediating plant-microbe interactions. However, the mechanisms by which flavonoids are perceived and trigger downstream signaling events in microbes remain largely unknown. In this study, we characterized AefR, a flavonoid-sensing transcriptional regulator from *Pseudomonas fluorescens* 2P24, a plant growth-promoting rhizobacterium (PGPR) known for its biocontrol properties. AefR was found to repress the expression of the mexEF-oprN efflux pump, which putatively exports N-acylhomoserine lactones (AHLs). This repression attenuates the PcoR/PcoI quorum-sensing system, leading to decreased production of the antibiotic mupirocin in *P. fluorescens* 2P24. Furthermore, quantitative proteomic analysis revealed that the PcoR/PcoI quorum-sensing system regulates a diverse range of physiological processes, including mupirocin production and denitrification. Collectively, these findings demonstrate a quorum-quenching role of flavonoids in a PGPR strain, establishing that flavonoids can disrupt quorum-sensing by enhancing the efflux of quorum-sensing signaling molecules. These findings have practical implications for the development of sustainable biocontrol strategies, where leveraging natural plant-microbe interactions could enhance the suppression of plant pathogens without the use of synthetic chemicals.

**IMPORTANCE:**

Flavonoids are key mediators of plant-microbe interactions; however, their role in regulating microbial signaling remains poorly understood. This study identifies AefR as a flavonoid-sensing regulator in *Pseudomonas fluorescens* 2P24, revealing a novel quorum-quenching mechanism where flavonoids enhance the efflux of quorum-sensing signals. These findings shed light on the molecular basis of flavonoid-mediated microbial regulation and offer new strategies for sustainable plant health management.

## INTRODUCTION

Flavonoids, a diverse class of plant secondary metabolites, play a pivotal role in mediating plant-microbe interactions in the rhizosphere (1, 2). These compounds are secreted by plant roots and serve as chemical signals that shape the composition and activity of the surrounding microbial community. Flavonoids can promote the recruitment of beneficial microbes, such as nitrogen-fixing rhizobia and plant growth-promoting rhizobacteria (PGPR), while simultaneously suppressing pathogenic microorganisms (3, 4). Additionally, flavonoids have been implicated in regulating bacterial behaviors, including biofilm formation, motility, and quorum sensing (QS) (5, 6). Despite their well-established roles in rhizobia-legume symbiosis, the molecular mechanisms through which flavonoids influence quorum sensing and biocontrol activity in PGPR remain largely unexplored.

Plant growth-promoting rhizobacteria (PGPR) are essential for sustainable agriculture due to their ability to promote plant growth and protect crops from soil-borne pathogens (7). These beneficial microbes employ diverse mechanisms to combat phytopathogens, such as competitive root colonization and the production of secondary metabolites that suppress harmful fungi and bacteria (8). Among PGPR, *Pseudomonas fluorescens* 2P24 is a widely studied strain known for its strong biocontrol activity and effective root colonization (9). By producing a range of secondary metabolites, including the antibiotic mupirocin, *P. fluorescens* 2P24 plays a crucial role in protecting crops from disease and reducing the need for chemical pesticides (10).

Quorum sensing is a bacterial communication system that coordinates gene expression in response to population density, enabling bacteria to synchronize collective behaviors such as biofilm formation, virulence, and the production of antimicrobial compounds. In gram-negative bacteria, quorum sensing is primarily mediated by N-acylhomoserine lactones (AHLs), which are synthesized by LuxI-like enzymes and recognized by LuxR-like receptors (11). When AHLs concentrations exceed a critical threshold, they bind to LuxR receptors, triggering a positive feedback loop that amplifies quorum sensing-regulated gene expression (12). In *P. fluorescens* 2P24, this system is governed by the PcoI/PcoR signaling system, where PcoI serves as the AHL-producing enzyme, and PcoR functions as the transcriptional regulator. The PcoI/PcoR system plays a critical role in regulating the disease-suppressive activities of strain 2P24, making it a key component of its biocontrol mechanisms (9).

Despite its critical role in interspecies communication, the QS system can be tuned down through multiple mechanisms, a phenomenon known as quorum quenching (13). One common mechanism is the enzymatic degradation of quorum-sensing signals by quorum-quenching enzymes, such as lactonases and acylases, which disrupt the accumulation of signaling molecules (14, 15). Certain chemicals, known as QS inhibitors, can interfere with quorum sensing by binding to QS receptors. Recently, resistance-nodulation-division (RND) efflux pumps such as MexAB-OprM and MexEF-OprN in *Pseudomonas* can export quorum-sensing molecules or their precursors, thereby reducing their intracellular levels and attenuating QS activity (16–19). These mechanisms highlight the dynamic nature of QS regulation and its susceptibility to environmental influences, offering potential avenues for controlling bacterial behavior in natural and agricultural settings.

In this study, we identified AefR, a flavonoid-sensing transcriptional regulator that controls the expression of the RND efflux pump MexEF-OprN, thereby interfering with the PcoR/PcoI QS system and antibiotic production. Additionally, we demonstrated that the PcoR/PcoI QS system regulates a broad range of physiological processes, including the biosynthesis of mupirocin. These findings provide new insights into the molecular mechanisms of flavonoid-mediated plant-microbe interactions and suggest potential strategies for improving biocontrol efficacy in sustainable agriculture.

## RESULTS

### AefR was a transcriptional repressor of efflux pump *mexEF-oprN*

Cofitness analysis has been shown to be a valuable tool for predicting gene functions (20, 21). In our study, we analyzed the cofitness data for the *P. fluorescens* FW300-N2E2 strain and identified that the EmhR homolog, which is known to be involved in antibiotic resistance, exhibited high cofitness values with Pf6N2E2_5647 (22, 23). This gene is a homolog of AefR, which has been previously implicated in regulating several virulence-associated traits in *Pseudomonas syringae* pv. syringae strain B728a (24). This suggests a potential role for AefR in modulating antibiotic resistance. To test this hypothesis, we measured the minimal inhibitory concentrations (MICs) of several antibiotics in wild-type *P. fluorescens* 2P24 and its Δ*aefR* mutant. Notably, the Δ*aefR* mutant showed increased resistance to kanamycin, chloramphenicol, and lomefloxacin compared with the wild-type strain (Table 1).

**TABLE 1:** MICs of wild-type strain and its derivatives

Mg/L	Ampicillin	Kanamycin	Chloramphenicol	Tetracycline	Gentamicin	Lomefloxacin
Wild type	512	4	128	16	8	2
Δ*aefR*	512	8	512	16	8	16
Δ*aefR* Δ*mexEF-oprN*	512	4	128	16	8	2

**Fig 1:**
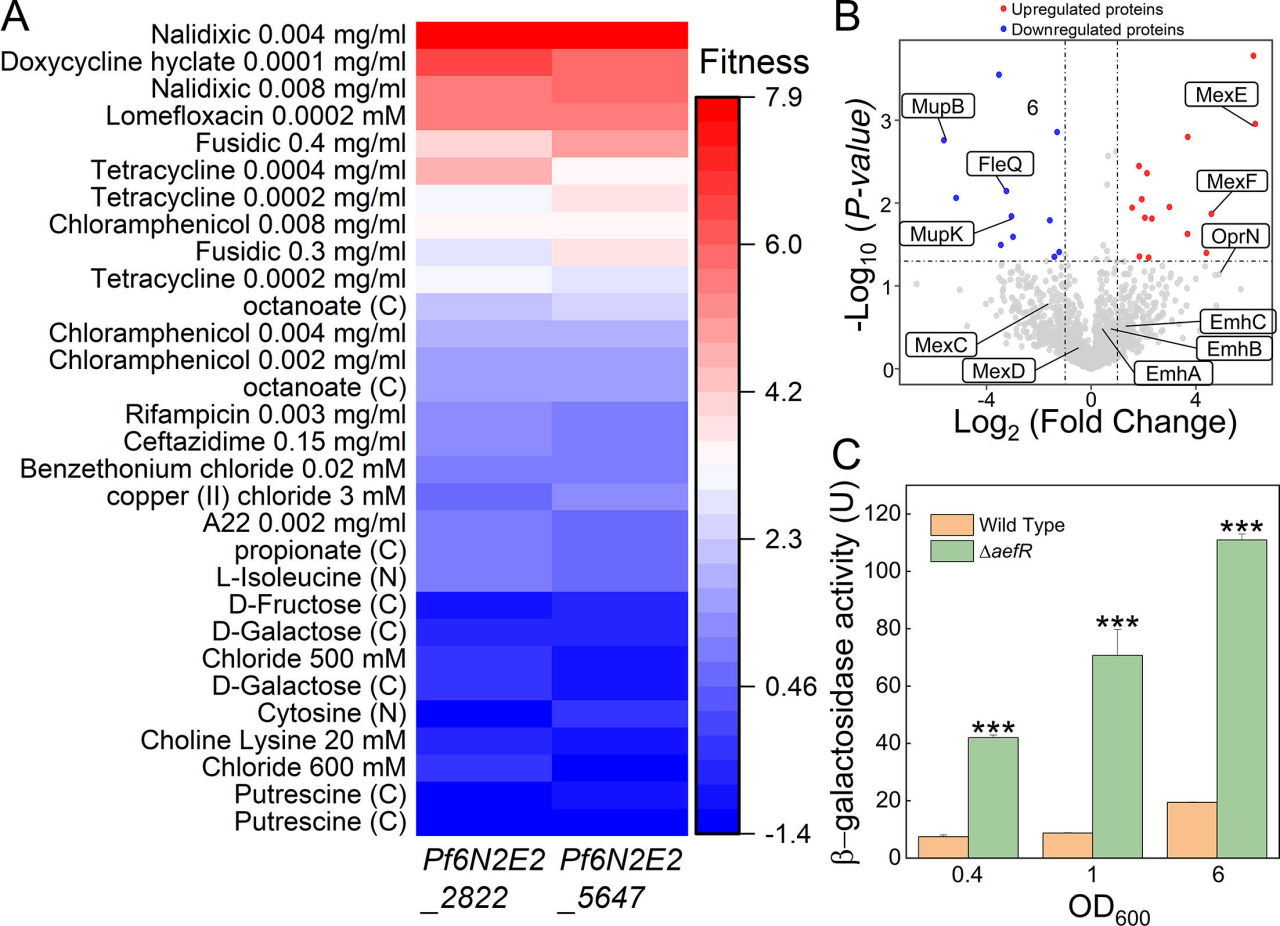
AefR was a transcriptional repressor of efflux pump *mexEF-oprN*. (**A**) Cofitness analysis of the orthologs of emhR *Pf6N2E2_2822* and aefR *Pf6N2E2_5647*. (**B**) Volcano plots showed the differentially expressed proteins in the Δ*aefR* strain compared with the wild-type *P. fluorescens* 2p24 strain. For each protein, the −log_10_ (*P*-value) is plotted against its log_2_ (fold change). Proteins upregulated (*P* < 0.05, fold change >2) in the Δ*aefR* strain are colored in red, whereas proteins downregulated (*P* < 0.05, fold change < −2) are colored in blue. (**C**) The β-galactosidase activities of pRG970-p*mexEF-oprN* were measured in the wild-type and Δ*aefR* strains at different OD_600_. Error bars denote standard deviation (*n* = 4). Student’s t test was used, *P* < 0.05 was displayed as *, *P* < 0.01 was displayed as **, *P* < 0.001 was displayed as ***.

Building on the observed role of AefR in antibiotic resistance, we conducted quantitative proteomic profiling of both the wild-type and Δ*aefR* strains. A total of 2,259 proteins were identified with a 1% false discovery rate (FDR). Applying an inclusion criterion of a greater than 2-fold change and a *P*-value of less than 0.05, we identified 16 upregulated and 12 downregulated proteins in the Δ*aefR* strain (Table S1). Strikingly, MexE and MexF, components of the MexEF-OprN efflux pump, were upregulated by approximately 44-fold and 31-fold, respectively, in the Δ*aefR* strain, whereas other efflux pumps, including MexC, MexD, EmhA, EmhB, and EmhC, did not show significant changes. Disruption of *mexEF-oprN* in the Δ*aefR* background restored antibiotic resistance to wild-type levels, demonstrating that AefR suppresses the expression of *mexEF-oprN* and thus modulates resistance to kanamycin, chloramphenicol, and lomefloxacin (Table 1). These results indicate that AefR represses *mexEF-oprN* expression, leading to enhanced resistance to these antibiotics in the Δ*aefR* mutant. To further validate these findings, we performed β-galactosidase reporter assays, which revealed significantly higher *mexEF-oprN* expression in the Δ*aefR* strain compared with the wild-type strain, further confirming that AefR directly represses the transcription of *mexEF-oprN*. Together, these findings establish a regulatory role for AefR in modulating antibiotic resistance through its effect on MexEF-OprN expression in *P. fluorescens* (Fig. 1C).

Moreover, MupB and MupK, key components of the mupirocin biosynthesis gene cluster (25, 26), were downregulated approximately 48-fold and 7-fold, respectively, compared with the wild-type strain, indicating that AefR was involved in the regulation of mupirocin production (Fig. 1B). It has been proven that mupirocin production in *P. fluorescens* was positively regulated by the quorum-sensing system in *P. fluorescens* NCIMB 10586 (27). Hence, we hypothesized that AefR could regulate mupirocin production by modulating quorum sensing.

### AefR regulated the production of mupirocin of *P. fluorescens* by modulating the quorum-sensing system

**Fig 2:**
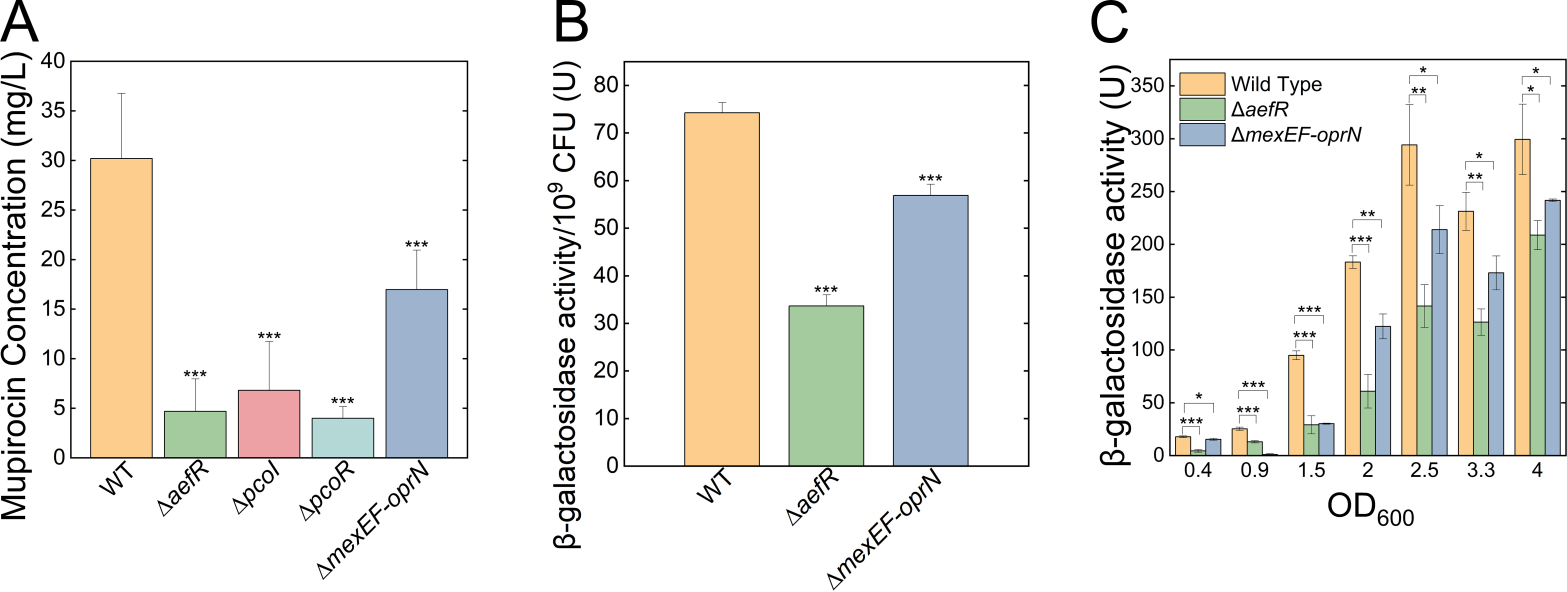
AefR regulated the production of mupirocin of *P. fluorescens* by the quorum-sensing system. (**A**) Mupirocin production assays. The levels of mupirocin production were measured in different strains at 48 h using the HPLC method. Error bars denote standard deviation (*n* = 3). Student’s t test was used, *P*-value < 0.05 was displayed as *, *P*-value < 0.01 was displayed as **, and *P*-value < 0.001 was displayed as ***. (**B**) Detection of the AHL signals reveals the accumulation of wild-type 2P24 strain and its derivatives in *A. tumefaciens* NTL4 (pZLR4). The AHL signal molecules were extracted from wild-type 2P24, Δ*aefR,* and Δ*mexEF-oprN* cultures and incubated with *A. tumefaciens* NTL4 (pZLR4). Then, the β-gal activities were detected to represent the production of AHLs in each strain. (**C**) The β-galactosidase activities of pRG970-p*pcoI* were measured in the wild-type 2P24, Δ*aefR,* and Δ*mexEF-oprN* strains at different OD_600_.

The proteomic data implied that AefR could regulate the production of mupirocin, and this prompted us to compare mupirocin production between Δ*aefR* and wild-type. The results showed that mupirocin production was significantly reduced in the Δ*aefR* compared with the wild-type strain (Fig. 2A). To investigate the role of AefR in regulating the quorum-sensing system in *P. fluorescens*, we conducted assays to measure N-acyl homoserine lactones (AHLs) production and β-galactosidase activity of the *pcoI* promoter fusion construct (*pRG970-ppcoI*). The AHLs production assays were carried out with biosensor strain *Agrobacterium tumefaciens* NTL4 (pZLR4), which allows the detection of multiple AHLs (28). The results revealed reduced AHLs production in the Δ*aefR* compared with the wild-type, indicating that AefR positively influenced AHLs production (Fig. 2B). Using the *lacZ* reporter fusion of the *pcoI* promoter, we further demonstrated that *pcoI* operon expression was significantly lower in the Δ*aefR* compared with the wild-type strain at different growth stages (Fig. 2C). Taken together, our findings demonstrated that AefR played crucial roles in regulating mupirocin production through their influence on the quorum-sensing system in *P. fluorescens*.

### MexEF-OprN affects the quorum-sensing system by regulating the expression of the quorum-quenching AHL hydrolase *mupX* gene and the AHL acylase *pvdQ* gene

**Fig 3:**
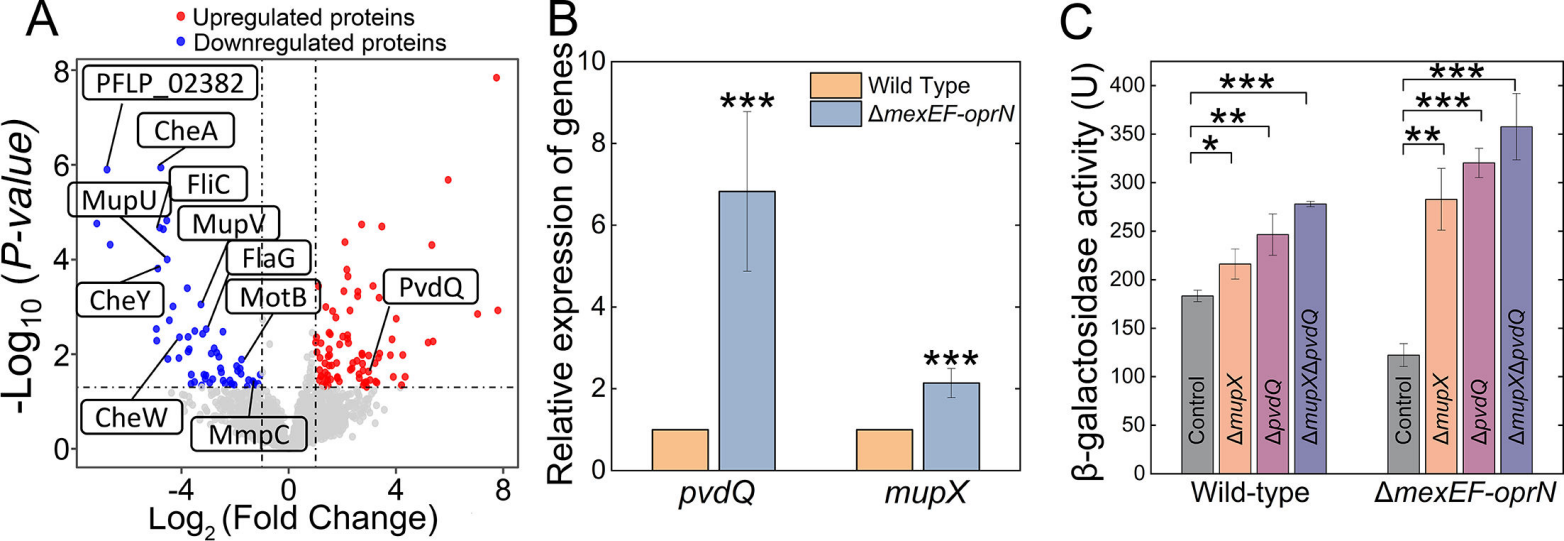
MexEF-OprN influenced the expression of orthologs of quorum-quenching AHL hydrolase *mupX* gene and AHL acylase *pvdQ* gene. (**A**) Volcano plots showed the differentially expressed proteins in the Δ*mexEF-oprN* strain compared with the wild-type *P. fluorescens* strain. For each protein, the −log_10_ (*P*-value) is plotted against its log_2_ (fold change). Proteins upregulated (*P* < 0.05, fold change >2) in the Δ*mexEF-oprN* strain are colored in red, whereas proteins downregulated (*P* < 0.05, fold change < −2) are colored in blue. (**B**) The transcription levels of *pvdQ* and *mupX* orthologs were measured in wild-type and Δ*mexEF-oprN* strain via qRT-PCR assays. Error bars denote standard deviation (*n* = 4). Student’s t test was used, *P* < 0.05 was displayed as *, *P* < 0.01 was displayed as **, and *P* < 0.001 was displayed as ***. (**C**) The β-galactosidase activities of pRG970-p*pcoI* were measured in the wild-type Δ*mexEF-oprN* strain with mutation of *mupX* and *pvdQ*. Error bars denote standard deviation (*n* = 3). Student’s t test was used, *P*-value < 0.05 was displayed as *, *P* < 0.01 was displayed as **, and *P*-value < 0.001 was displayed as ***.

Since AefR acted as a repressor of MexEF-OprN efflux pump, and this pump has been implicated in the negative regulation of quorum-sensing systems in *P. syringae* pv. *tabaci* 6605 (16). We investigated if AefR influenced the quorum-sensing system in *P. fluorescens* by regulating the expression of *mexEF-oprN*. However, in our work, deletion of *mexEF-oprN* unexpectedly inhibited quorum-sensing activity, leading to reduced mupirocin production (Fig. 2A through C). To explore the underlying mechanism, proteomic analysis was conducted to identify differentially expressed proteins in the Δ*mexEF-oprN* strain compared with the wild type. In the *mexEF-oprN* mutant, MupV, MupU and MmpC within the mupirocin biosynthesis gene cluster were reduced by approximately 12-fold, 22-fold, and 2-fold, respectively, compared with the wild-type (Fig. 3A). Moreover, the analysis revealed that the expression of PvdQ, an AHL acylase, was upregulated 5-fold in the Δ*mexEF-oprN* strain (Fig. 3A; Table S2). PvdQ is known for its quorum-quenching activity in *P. aeruginosa* by degrading AHLs (15, 29). Hence overexpression of the *pvdQ* gene could be a potential reason underlying the decrease of AHLs in the Δ*mexEF-oprN* strain. Additionally, the ability of MupX to degrade AHLs across a wide range of chain lengths (C4–C14), with or without a 3-oxo substituent, has been previously documented (27). Quantitative real-time PCR (qRT-PCR) further confirmed elevated transcription levels of *pvdQ* and *mupX* in the Δ*mexEF-oprN* strain, showing 2-fold to 7-fold increases compared with the wild type (Fig. 3B). Based on these findings, we hypothesized that the absence of MexEF-OprN caused an accumulation of intracellular AHLs, which in turn triggered the upregulation of AHL-degrading enzymes like PvdQ and MupX, thereby inhibiting quorum sensing. To test this, in-frame deletion mutants of *mupX* and *pvdQ* were constructed. Deletion of either gene led to a significant increase in *pcoI* operon expression in both the wild-type and Δ*mexEF-oprN* strains (Fig. 3C). These results suggest that the quorum-sensing inhibition observed in the Δ*mexEF-oprN* strain is primarily due to the increased expression of *pvdQ* and *mupX*, which mediate enhanced degradation of AHLs, disrupting quorum-sensing signaling pathways.

### Proteomics revealed that PcoI/PcoR quorum-sensing system regulated mupirocin production in *P. fluorescens*

**Fig 4:**
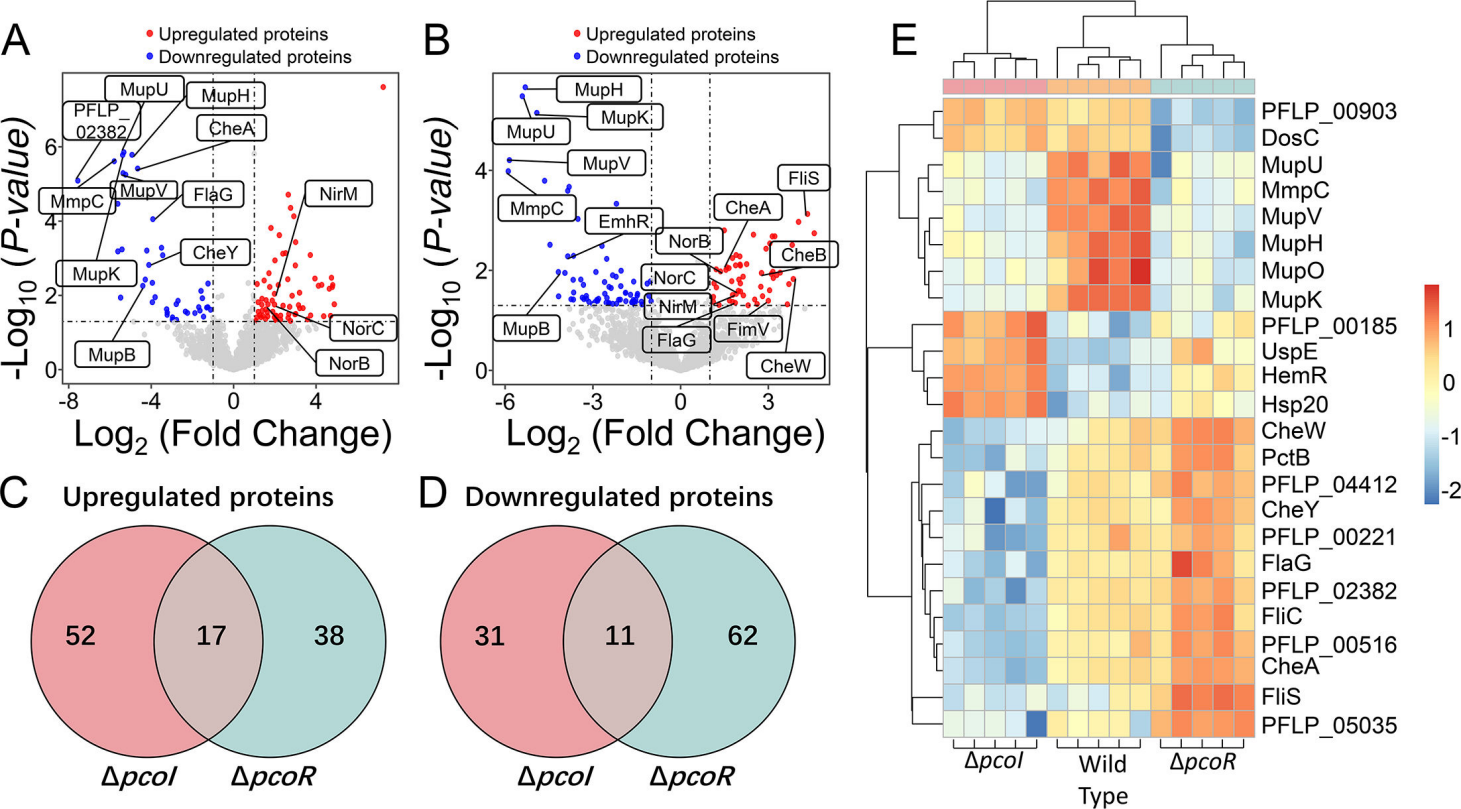
PcoI/PcoR quorum-sensing system regulated multiple physiological behaviors in *P. fluorescens*. Volcano plots showed the differentially expressed proteins in the Δ*pcoI* (**A**) and Δ*pcoR* (**B**) strains compared with the wild-type *P. fluorescens* 2P24 strain. For each protein, the −log_10_ (*P*-value) is plotted against its log_2_ (fold change). Proteins up-regulated (*P*-value < 0.05, fold change >2) are colored in red while proteins down-regulated (*P* < 0.05, fold change < −2) are colored in blue. Venn diagrams were drawn to show the upregulated (**C**) and downregulated proteins (**D**) by more than 2-fold (*t*-test, *P*-value < 0.05) in the Δ*pcoI* and Δ*pcoR* strains. (**E**) The heat map of the differentially expressed proteins in wild-type *P. ﬂuorescens* 2P24 and its derivatives. Two-way ANOVA (FDR 5%) was performed to identify 24 proteins showing signiﬁcant expression changes between any two of the strains. The five rectangles corresponded to gene expression levels in five independent samples. Proteins were colored according to their normalized expression levels. A subsequent unsupervised hierarchical clustering revealed four clusters of co-regulated proteins showing different expression patterns in response to Δ*pcoI* and Δ*pcoR*.

To further explore the regulatory networks of this quorum-sensing system, we performed label-free quantification proteomic analysis on the wild-type *P. fluorescens* 2P24 strain and its derivatives, including the Δ*pcoI* and Δ*pcoR* mutants. This analysis aimed to identify downstream targets of the PcoR/PcoI system. In total, 3,196 proteins were identified with 1% FDR and an inclusion criterion of greater than 2-fold change, and a *P*-value less than 0.05 was applied to screen the differentially expressed proteins (DEPs), resulting in 69 upregulated and 42 downregulated proteins in Δ*pcoI* strain (Fig. 4A) and 55 upregulated and 73 downregulated proteins in Δ*pcoR* strain (Fig. 4B). Among these, 17 proteins showed increased expression in both Δ*pcoI* and Δ*pcoR* mutants (Fig. 4C). Notably, these included nitrogen metabolism-related proteins such as NirM, NorB, and NorC, which exhibited 2.4-fold to 3.5-fold and 2.4-fold to 7-fold increases in Δ*pcoI* and Δ*pcoR* mutants, respectively (Fig. 4A and B). These findings suggest that PcoI and PcoR negatively regulate the denitrification process, potentially enhancing anaerobic survival (30). Conversely, 11 proteins were consistently downregulated in both mutants, primarily enzymes involved in the biosynthesis of the antibiotic mupirocin, such as MupK, MupB, MupH, MmpC, MupV, and MupU (Fig. 4A and B). This indicates that the PcoR/PcoI quorum-sensing system positively regulates mupirocin production in *P. fluorescens*. Mupirocin production in Δ*pcoI* and Δ*pcoR* strains was significantly decreased compared with the wild-type strain (Fig. 2A). Interestingly, the proteomic data also revealed opposing roles for PcoI and PcoR in regulating bacterial chemotaxis and flagella-related motility. Proteins involved in chemotaxis, including CheA, CheB, and CheW, were upregulated by 2.8-fold to 13-fold in the Δ*pcoR* strain, but CheA and CheY were downregulated by 18-fold to 180-fold in the Δ*pcoI* strain (Fig. 4E). In contrast, flagella-related proteins such as FlaG, FliC, and FliS were significantly upregulated in the Δ*pcoR* strain but downregulated in the Δ*pcoI* strain (Tables S3 and S4). In summary, proteomic analysis demonstrated that the PcoR/PcoI quorum-sensing system positively regulates mupirocin biosynthesis and negatively regulates the denitrification process. Additionally, PcoI promotes bacterial chemotaxis and motility, whereas PcoR acts as a repressor of these processes. These findings illuminate the complex and multifaceted regulatory roles of PcoR and PcoI in *P. fluorescens*.

### AefR is a flavonoid-sensing transcriptional regulator

**Fig 5:**
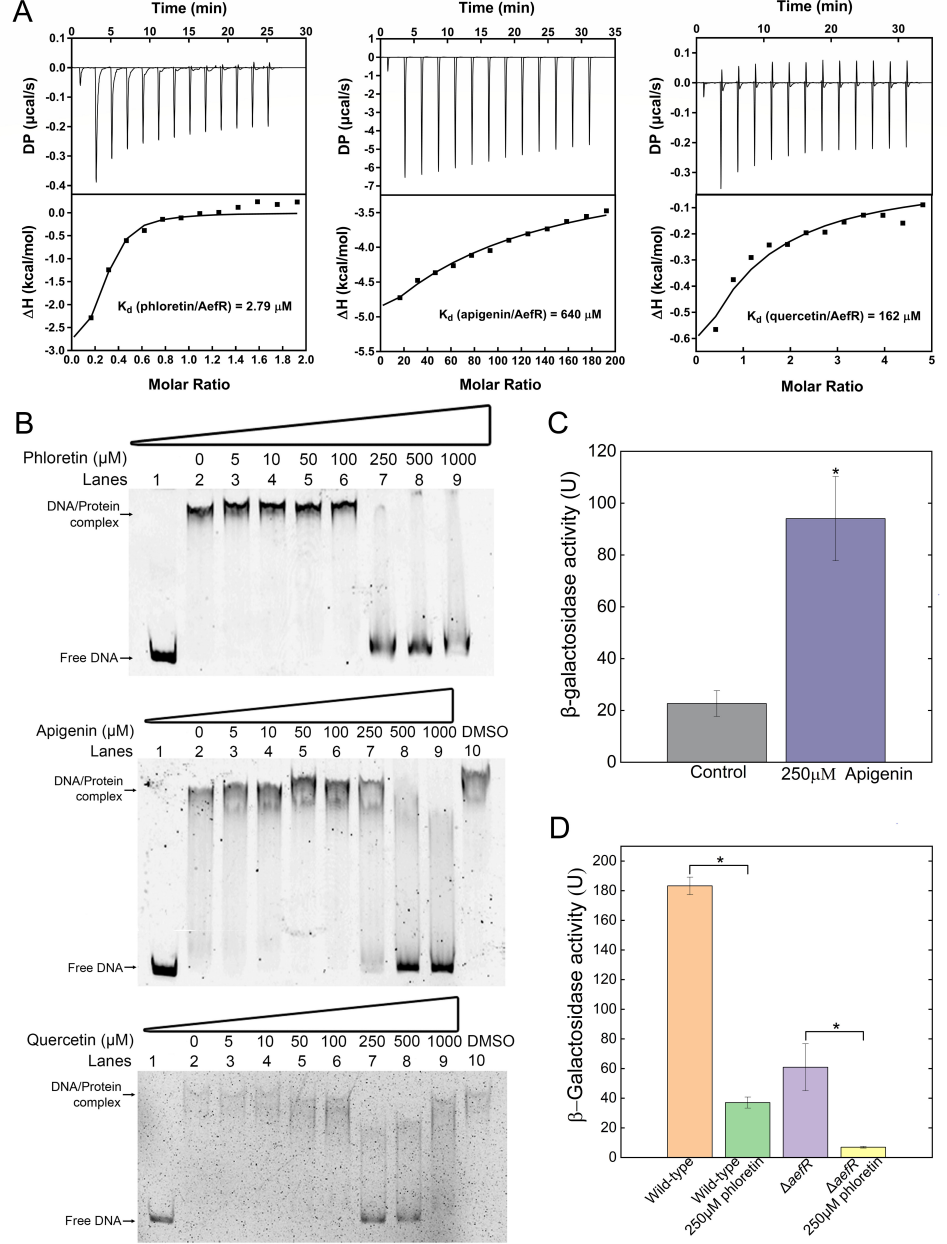
AefR could respond to flavonoids, and phloretin downregulate the expression of PcoI. (**A**) The isothermal titration calorimetry (ITC) assay was carried out to determine the interaction between His^6^-AefR and phloretin, apigenin, and quercetin, respectively. Data were analyzed using the MicroCal PEAQ-ITC software and the dissociation constant kd was shown. (**B**) EMSAs were used to determine the effect of flavonoids on the interaction between AefR and p*mexEF-oprN*. The interaction between AefR and p*mexEF-oprN* was significantly reduced in the presence of flavonoids compared with the control group. (**C**) The β-galactosidase activities of pRG970-p*mexEF-oprN* were measured in the wild-type *P. fluorescens* 2p24 strain treated with DMSO, 250 µM apigenin. Error bars denote standard deviation (*n* = 3). Student’s t test was used, *P* < 0.05 was displayed as *, *P* < 0.01 was displayed as **, and *P* < 0.001 was displayed as ***. (**D**) The β-galactosidase activities of pRG970-p*pcoI* were measured in the wild-type *P. fluorescens* strain and Δ*aefR* strain in the presence of 250 µM phloretin. Error bars denote standard deviation (*n* = 3). Student’s t test was used, *P*-value < 0.05 was displayed as *, *P*-value < 0.01 was displayed as **, and *P*-value < 0.001 was displayed as ***.

Since TetR-family regulators typically sense specific ligands, we screened a series of chemicals and found that AefR binds to the plant-derived flavonoids including phloretin, quercetin, and apigenin with dissociation constants of 2.79, 640, and 162 µM, respectively (Fig. 5A). Furthermore, we demonstrated that AefR binds to the upstream regulatory sequence of the mexEF-oprN operon in a concentration-dependent manner (Fig. S1). Notably, addition of flavonoids—phloretin, apigenin, and quercetin—at concentrations of 250, 500, and 250 µM, respectively, disrupted this interaction, indicating that flavonoids can influence the transcriptional regulatory networks mediated by AefR (Fig. 5B). Since flavonoids have been shown to disrupt quorum sensing in *P. aeruginosa*, we speculated that they could similarly modulate quorum sensing in the PGPR *P. fluorescens*. To test this, we conducted β-galactosidase activity assays to assess the effects of flavonoids on the expression of *pcoI*, a key quorum sensing-related gene, in the wild-type 2P24 strain. Our results revealed that apigenin increased *mexEF-oprN* expression (Fig. 5C), which promoted multidrug resistance in *P. fluorescens* 2P24 (Table 2). In contrast, phloretin effectively suppressed *pcoI* expression in both the wild-type and *aefR* mutant strains (Fig. 5D), suggesting that phloretin represses *pcoI* through mechanisms independent of MexEF-OprN. Although structurally similar, flavonoids appear to modulate bacterial signaling pathways through distinct mechanisms. These findings highlight the nuanced role of flavonoids in influencing both multidrug resistance and quorum-sensing regulation in *P. fluorescens*.

**TABLE 2:** MICs of wild-type strain and Δ*aefR* strain treated with flavonoids^*a*^

Mg/L	Ampicillin	Kanamycin	Chloramphenicol	Tetracycline	Gentamicin	Lomefloxacin
Wild type	512	4	128	16	8	2
Wild type + A	512	8	512	16	8	8
Wild type + *P*	512	4	128	16	8	2
Wild type + Q	512	4	128	16	8	2
Δ*aefR*	512	8	512	16	8	16
Δ*aefR* + A	512	8	512	16	8	16
Δ*aefR* + *P*	512	8	512	16	8	16
Δ*aefR* + Q	512	8	512	16	8	16

^*a*^ A: Apigenin, P: Phloretin, Q: Quercetin.

## DISCUSSION

Flavonoids are well-established signaling molecules that facilitate interactions between plants and rhizosphere microorganisms (5, 31). Early *in vitro* studies indicated that flavonoids can induce the expression of rhizobial nod genes and act as chemo-attractants, increasing the concentration of rhizobia on root surfaces (32). Additionally, flavonoids play a key role in mitigating bacterial pathogenicity by acting as quorum-sensing inhibitors. For instance, in a mutant strain of *P. syringae* DC3000 lacking the MexAB-OprM transporter, flavonoids were found to disrupt the GacS/GacA two-component system, which influences the production of microbial flagella and the type III secretion system (33). In *P. aeruginosa*, flavonoids specifically block quorum sensing by antagonizing the autoinducer-binding receptors LasR and RhlR (3). Recently, several flavonoids have been shown to inhibit biofilm formation in *P. aeruginosa*, further demonstrating their potential role in controlling *P. aeruginosa* pathogenicity (34, 35). In this work, we investigated the mechanisms of flavonoids influencing the quorum-sensing system in *P. fluorescens*. Although both phloretin and apigenin disrupted the interaction between AefR and its target DNA, their downstream effects differed. Apigenin reversed AefR’s repression of *mexEF-oprN* expression, whereas phloretin reduced *pcoI* expression, suggesting distinct regulatory outcomes despite structural similarities. The ability of flavonoids to fine-tune QS systems highlights their critical role in maintaining a balanced rhizosphere microbiome, promoting plant health, and suppressing pathogens (36). Leveraging flavonoids in agricultural systems offers a natural and sustainable strategy for enhancing biocontrol effectiveness and supporting plant resilience against environmental challenges (37). Our findings underscore how subtle structural variations among flavonoids can fine-tune bacterial signaling responses, demonstrating the nuanced impact of environmental compounds on regulatory networks.

Quorum-sensing systems are essential for the biocontrol efficacy of plant growth-promoting rhizobacteria (PGPRs), influencing processes such as colonization, nodulation, signal transduction, and interactions with other microorganisms (38–40). These systems are governed by intricate regulatory networks involving Sigma factor *rpoS*, the two-component system *gacA/gacS*, TetR-family regulators, and multidrug efflux pumps (41–44). Notably, multidrug efflux pumps play dual roles in antibiotic resistance and gene expression regulation (45). For instance, the EmhABC efflux pump negatively regulates the production of the secondary metabolite 2,4-diacetylphloroglucinol (2,4-DAPG) in *P. fluorescens* 2P24 (22). In *P. aeruginosa*, efflux pumps contribute to quorum sensing regulation by expelling PQS signal precursors, modulating intracellular signal concentrations (46). Efflux pumps like MexAB-OprM and MexEF-OprN have been implicated in the negative regulation of quorum-sensing systems by reducing the intracellular concentration of key signal molecules. For example, MexEF-OprN expels 3-oxo-C12-HSL in *P. aeruginosa*, leading to decreased intracellular quorum-sensing signaling (17, 44). Similarly, overexpression of *MexEF-OprN* reduces the synthesis of C4-HSL via repression of the *rhlI* gene in *P. aeruginosa* (18). Our study revealed that in *P. fluorescens*, MexEF-OprN modulates the quorum-sensing system by likely expelling AHL signal molecules, altering intracellular AHLs concentrations, and influencing the expression of AHL-degrading genes such as *pvdQ* and *mupX*. These findings suggest that efflux pump inhibitors could serve as a novel approach to modulate QS-mediated bacterial communication.

**Fig 6:**
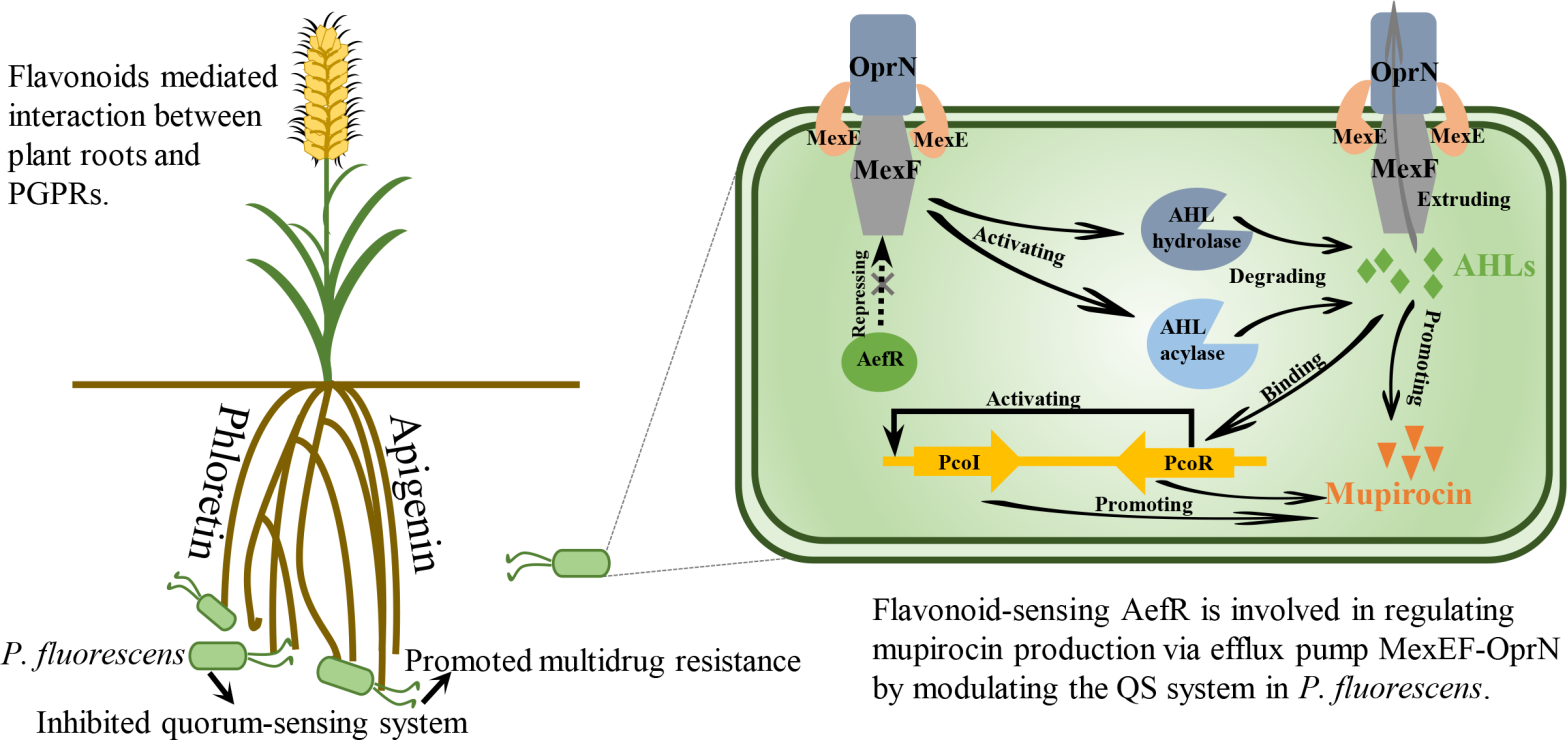
Graphic illustration of how AefR regulates quorum-sensing system and multidrug resistance by responding to flavonoids in *P. ﬂuorescens* 2p24. AefR could positively regulate quorum-sensing systems via modulating expression of efflux pump MexEF-OprN, which could pump quorum-sensing signal molecules AHLs out of the bacteria. With elevated flavonoid concentration in the rhizosphere, *P. fluorescens* 2P24 promotes multidrug resistance and inhibits quorum sensing, enhancing root colonization potential.

In summary, we identified AefR, a TetR-family regulator, as a receptor responsive to flavonoids that positively regulates the quorum-sensing system by inhibiting the expression of the MexEF-OprN efflux pump (Fig. 6). This regulatory interaction underscores AefR’s crucial role in modulating quorum-sensing pathways via control of the efflux pump, highlighting the efflux pump’s potential as a key regulator of quorum sensing.

## MATERIALS AND METHODS

### Bacterial strains and culture conditions

For molecular cloning, *Escherichia coli* DH5α (Stratagene, La Jolla, CA) was utilized, whereas *E. coli* BL21 (DE3) cells (Novagen, Madison, WI, USA) were used to express recombinant proteins (Table 3). Cultures of *E. coli* strains were grown in Luria-Bertani (LB) broth at 37°C with shaking at 200 rpm. *P. fluorescens* 2P24 and its derivative strains were cultured at 28°C with shaking at 200 rpm in liquid King’s B (KB) medium or on KB agar plates. When necessary, growth media were supplemented with selective agents and inducers, including ampicillin (50 mg/L), kanamycin sulfate (50 mg/L), chloramphenicol (25 mg/L), sucrose (10% wt/vol), isopropyl-β-D-thiogalactopyranoside (IPTG, 0.2 mM), and 5-bromo-4-chloro-3-indolyl-β-D-galactopyranoside (X-Gal, 40 mg/L).

**TABLE 3:** Table of strains in this study

Strain name	Plasmid^r^	Source
Wild type	None	This study
Δ*aefR*	None	This study
Δ*pcoI*	None	This study
Δ*pcoR*	None	This study
Δ*mexEF-oprN*	None	This study
Δ*pvdQ*	None	This study
Δ*mupX*	None	This study
Δ*pvdQ*Δ*mupX*	None	This study
Δ*mexEF-oprN*Δ*pvdQ*Δ*mupX*	None	This study
WT_p*pcoI* Reporter	pRG970-p*pcoI*^Km^	This study
Δ*aefR*_p*pcoI* Reporter	pRG970-p*pcoI*^Km^	This study
Δ*mex*_p*pcoI* Reporter	pRG970-p*pcoI*^Km^	This study
WT_p*mexEF-oprN* Reporter	pRG970-p*mexEF-oprN*^Km^	This study
Δ*aefR*_p*mexEF-oprN* Reporter	pRG970-p*mexEF-oprN*^Km^	This study
*A. tumefaciens* NTL4	pZLR4^Gm^	(25)
*E. coli* DH5α	None	Stratagene, La Jolla, CA
*E. coli* BL21 (DE3)	None	Novagen, Madison, WI, USA
*E. coli* WM3064 (DE3)	None	This study

### Protein expression and purification

The *aefR* gene was amplified from genomic DNA, digested with *NdeI* and *XhoI* restriction enzymes, and cloned into a pET-28b-derived vector for overexpression in *E. coli* BL21 (DE3) cells. Protein expression was initiated by adding 0.2 mM IPTG to cultures at an optical density (600 nm) of 0.6–0.8, followed by incubation at 16°C for 20 h. Cells were harvested by centrifugation at 6,000 *g* for 10 min and resuspended in a buffer containing 20 mM Tris–HCl (pH 8.0), 500 mM NaCl, and 10 mM imidazole. The suspension was sonicated for 30 min to lyse the cells, and the lysate was clarified by centrifugation at 12,000 *g* for 30 min. The resulting supernatant was loaded onto a Ni-NTA affinity column, and the target protein was eluted using a buffer containing 20 mM Tris–HCl (pH 7.5), 100 mM NaCl, and 250 mM imidazole. The purified protein was concentrated at 20 mg/mL and stored at −80°C for future use.

### Quantification of mupirocin

Mupirocin quantification was conducted following a previously established method using high-performance liquid chromatography (HPLC) (47). *P. fluorescens* 2P24 and its derivatives were analyzed for mupirocin production. Samples were centrifuged at 12,000 *g* for 10 min, and the supernatant was subjected to HPLC analysis. The mobile phase consisted of acetonitrile and distilled water, each containing 0.1% acetic acid. An Agilent ZORBAX Eclipse XDB-C18 column (4.6 × 150 mm) was calibrated using 5% acetonitrile. A gradient elution was applied, increasing acetonitrile from 0% to 40% over 3 min, followed by a transition from 40% to 100% over the next 3 min, at a flow rate of 1 mL/min. Mupirocin was detected with a retention time of 7.2 min.

### Construction of in-frame deletion mutants of *P. fluorescens* 2p24

In-frame deletion mutants were constructed using a two-step homologous recombination method (48). Approximately 1 kb of the upstream and downstream regions of the target genes was amplified by PCR from *P. fluorescens* 2P24 genomic DNA using primers listed in Table S5. These fragments were digested with specific restriction enzymes and ligated into the suicide vector pK18mobsacB. The resulting plasmid was transformed into *E. coli* WM3064 and subsequently introduced into the recipient strain via conjugation. Mutants were selected on media containing 10% sucrose, and successful deletion was confirmed through PCR analysis following established protocols.

### Construction of transcriptional *lacZ* fusion and β-galactosidase assay

The promoter region of *mexEF-oprN* and *pcoI* was amplified and cloned into the reporter vector pRG970km by homologous recombination. The resulting plasmid was transformed into *E. coli* WM3064, and conjugation was carried out to transfer the plasmid to *P. fluorescens* 2P24 and its derivatives. For β-galactosidase assays, bacteria were cultured in KB at 28℃ overnight, supplied with appropriate antibiotics. The β-galactosidase activities were measured as described by Miller (49).

### Electrophoretic mobility shift assay (EMSA)

FAM-*mexE* was obtained by PCR amplification. The DNA fragments were added 50 nM and incubated at room temperature for 30 min with His-AefR protein in a total volume of 20 µL mixture. The mixture contained 50 mM Tris-HCl pH 7.5, 10 mM MgCl_2_, 10% (vol/vol) glycerol, 0.5 mM EDTA, and 50 mM KCl. Human serum albumin (HSA) was added at 3 µM concentration to prevent non-specific binding. After incubation, all 20 µL mixtures were added to 5 µL 50% (wt/vol) sucrose as loading buffer and subjected to 5% PAGE with 1× Tris borate-EDTA (TBE) buffer. Electrophoresis was performed at 80 V, 4°C in an ice-cold bath. For FAM-labeled probes, the images were acquired by a UVP BioSpectrum Imaging System (UVP, CA, USA).

### Isothermal titration calorimetry (ITC) assay

The dissociation constants (Kd) of phloretin and apigenin binding to AefR separately were determined by isothermal titration calorimetry (ITC). In addition, 5 mM phloretin and 20 mM apigenin dissolved in DMSO were prepared and diluted in Tris buffer (pH 7.5) at final concentrations of 500 µM and 2,000 µM, respectively. The protein AefR was diluted in Tris buffer (pH 7.5) at 50 µM or 2 µM concentration to perform ITC assay. Flavonoids titrated into Tris buffer continued AefR with 13 injections protocol being adjusted, the first drop is 0.4 µL and sustains 0.8 s, and the rest drops are 3 µL and sustain 6 s, drops are spaced 120 s apart. Flavonoids titrated into Tris buffer without protein AefR were performed as background, our data showed have erased the background.

### Total protein extraction and mass spectrometry (MS) analysis

Bacterial cells of wild-type *P. fluorescens* 2P24 and its derivatives were collected at the early stationary phase, lysed, reduced, and alkylated according to a modified protocol of Matthias Mann et al. For MS analysis, the peptides were resuspended in 0.1% formic acid (FA) and analyzed using an Orbitrap Fusion Lumos mass spectrometer (Thermo Scientific) coupled online to an EASY-nLC 1200 system in the data-dependent mode. Briefly, 4 µL of peptide sample was injected into a 15 cm long, 75 µm inner diameter capillary analytic column packed with C18 particles of 1.9 µm diameter. The mobile phases for the liquid chromatography (LC) include buffer A (0.1% FA) and buffer B (80% acetonitrile, 0.1% FA). The peptides were separated using a 90 min nonlinear gradient consisting of 5-35% buffer B for 60 min, 35%–80% buffer B for 20 min, and 100% buffer B for 10 min at a flow rate of 300 nL/min. The source voltage and current were set at 2.5 KV and 100 A, respectively. All MS measurements were performed in the positive ion mode and acquired across the mass range of 300–1800 m/z. The raw mass spectrometry files were analyzed by the software Proteomics Discovery, and MS/MS spectra were searched against the protein sequences of *P. fluorescens* 2P24. The methionine oxidation and cysteine carbamidomethylation were included as the variable modification and fixed modification, respectively. Other parameters were set up using the default values, and the FDR was set to 0.01 for both peptide and protein identifications. The extracted ion chromatograms (XICs) of identified peptides were used for the label-free quantification. Bioinformatic and statistical analyses were performed using software Perseus (50). Student’s *t* test was used to determine the significance of the differential expression of proteins between the wild-type strain and its derivatives. A *P* value < 0.05 was used as the cutoff for all statistical analyses.

## Data Availability

The mass spectrometry proteomics data have been deposited to the ProteomeXchange Consortium via the PRIDE partner repository with the data set identifier PXD052702 and PXD052703 (http://www.proteomexchange.org) (51, 52).

## SUPPLEMENTAL MATERIAL

Fig. S1 msystems.00915-24-s0001.pdf EMSA and DNase I footprinting were used to determine the interaction between His6-AefR and the promoter region of *mexEF-oprN* designated FAM-p*mexEF-oprN*.

Supplemental Sequencing Information msystems.00915-24-s0002.pdf Employing the Sanger sequencing method, it was identified that PFLP_3614 and PFLP_3613 would align to present the entirety of MupX, whereas PFLP_03091, PFLP_03092, and PFLP_03093 were aligned with PvdQ.

Table S1 msystems.00915-24-s0003.xlsx Up- and downregulated proteins in Δ*aefR* compared with wild-type *P. fluorescens* 2P24 strain.

Table S2 msystems.00915-24-s0004.xlsx Up- and downregulated proteins in Δ*mexEF-oprN* compared with wild-type *P. fluorescens* 2P24 strain.

Table S3 msystems.00915-24-s0005.xlsx Up- and downregulated proteins in Δ*pcoI* compared with wild-type *P. fluorescens* 2P24 strain.

Table S4 msystems.00915-24-s0006.xlsx Up- and downregulated proteins in Δ*pcoR* compared with wild-type *P. fluorescens* 2P24 strain.

Table S5 msystems.00915-24-s0007.xlsx Primers used in this study.

ASM does not own the copyrights to Supplemental Material that may be linked to, or accessed through, an article. The authors have granted ASM a non-exclusive, world-wide license to publish the Supplemental Material files. Please contact the corresponding author directly for reuse.
