# Supplementary material for: The flavonoid-sensing regulator AefR is involved in modulating quorum sensing through repressing the MexEF-OprN efflux pump in Pseudomonas fluorescens
Source: mSystems. 2025 Feb 27;10(3):e00915-24. doi: 10.1128/msystems.00915-24 (PMC11915855; doi:10.1128/msystems.00915-24)
Supplement: Fig. S1 — EMSA and DNase I footprinting were used to determine the interaction between His6-AefR and the promoter region of mexEF-oprN designated FAM-pmexEF-oprN. [file msystems.00915-24-s0001.pdf]

Supplementary Information

The flavonoid-sensing regulator AefR is involved in modulating quorum sensing through repressing the MexEF-OprN efflux pump in *Pseudomonas fluorescens*

Xiao-Quan Yu[b], Jian-Ting Han[a], Han-Zhong Feng[a], Jun Hou[a], Zhi-Ping Wang[b], Yong-Xing He[a*]

Table of Contents

Figures

Figure S1.---3

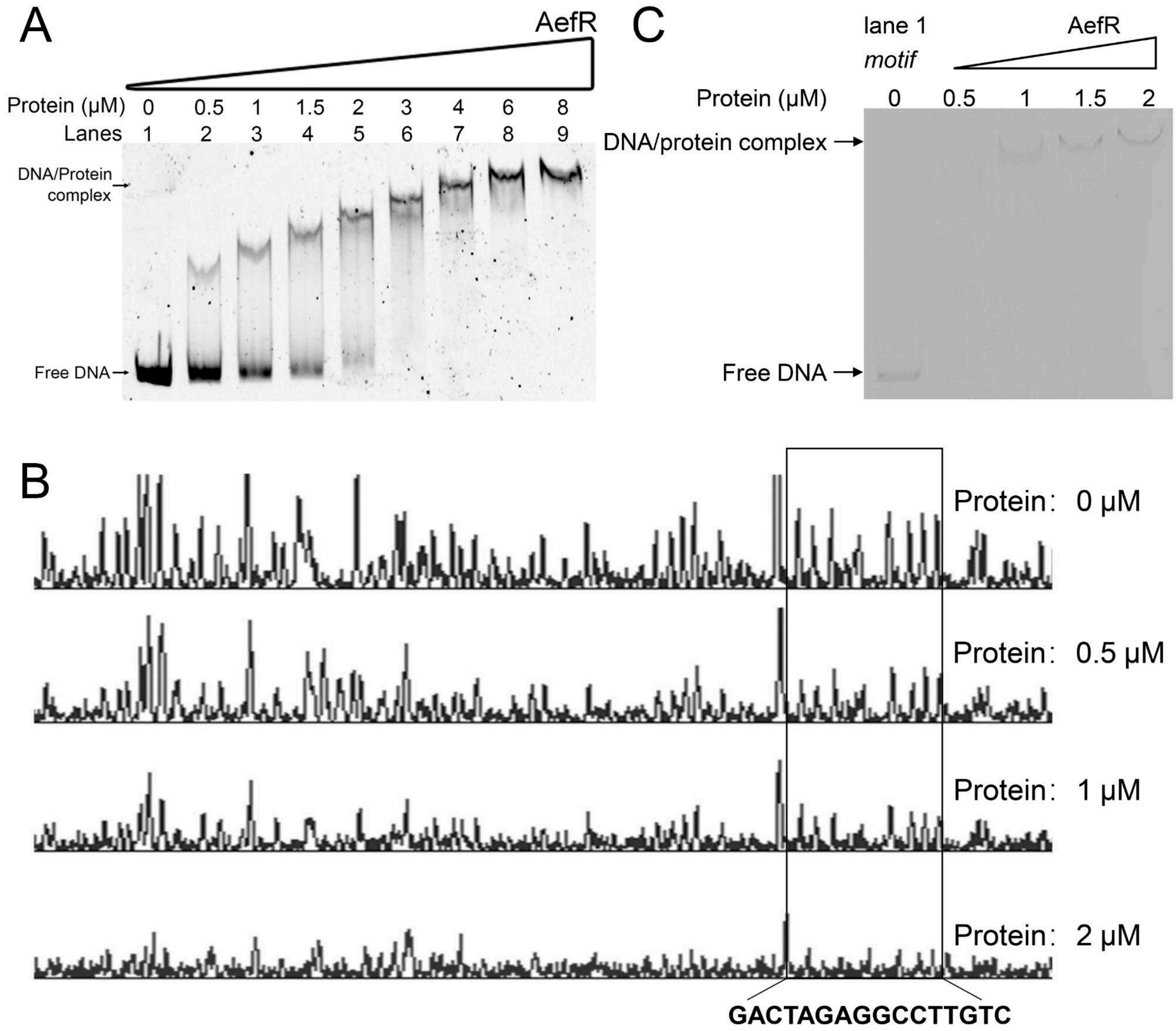

Figure S1. EMSA and DNase I footprinting were used to determine the interaction between His$_6$-AefR and the promoter region of *mexEF-oprN* designated as FAM-p*mexEF-oprN*. (A) The probe FAM-p*mexEF-oprN* was incubated with different concentrations of His$_6$-AefR (0.5-8 μM) or with buffer alone as a negative control (lane 1). (B) The DNase I footprinting analysis of FAM-p*mexEF-oprN* in the presence of 0 μM AefR protein (control), 0.5 μM AefR , 1 μM AefR and 2 μM AefR . The semi-palindromic region protected by AefR is boxed, and a sequencing map of p*mexEF-oprN* is shown at the bottom. (C) The probe FAM-*motif* was incubated with different concentrations of His$_6$-AefR (0.5-2 μM) or with buffer alone as a negative control (lane 1).
