# Supplementary material for: The flavonoid-sensing regulator AefR is involved in modulating quorum sensing through repressing the MexEF-OprN efflux pump in Pseudomonas fluorescens
Source: mSystems. 2025 Feb 27;10(3):e00915-24. doi: 10.1128/msystems.00915-24 (PMC11915855; doi:10.1128/msystems.00915-24)
Supplement: Supplemental Sequencing Information — Employing the Sanger sequencing method, it was identified that PFLP_3614 and PFLP_3613 would align to present the entirety of MupX, whereas PFLP_03091, PFLP_03092, and PFLP_03093 were aligned with PvdQ. [file msystems.00915-24-s0002.pdf]

Supplementary Sequencing Information

Employing the Sanger sequencing method, it was identified that PFLP_3614 and PFLP_3613 would align to present the entirety of MupX, whereas PFLP_03091, PFLP_03092, and PFLP_03093 were aligned with PvdQ. An illustration was showed as bellow and sequences of *pvdQ* and *mupX* were listed.

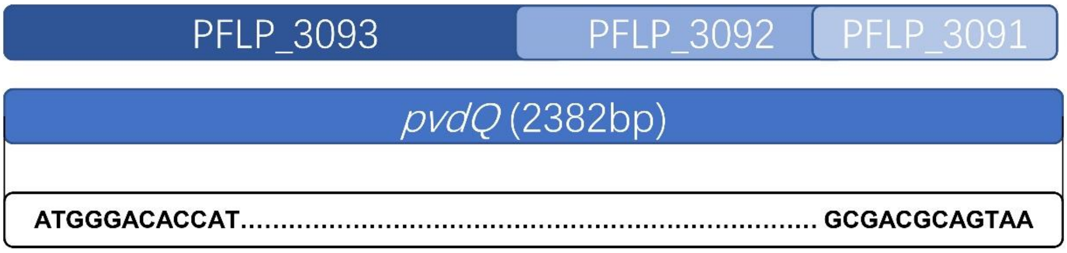

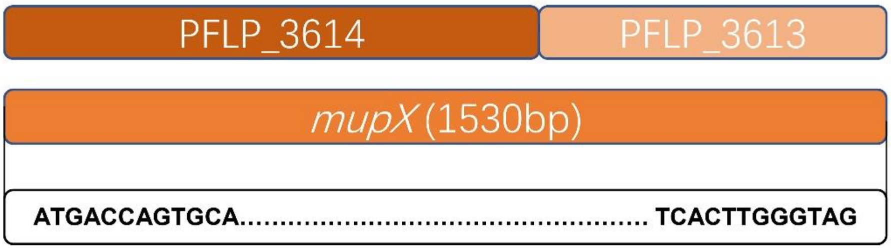

pvdQ Sequence:

ATGGGACACCATCACCATCACCATATGGTGAGAATGTCCGGGCAGTTATCGAGGATTGGCCTCGCAG
GCGCTTTTCTGGGGATCGCCCTCGGGCTCAGTCCCGTGGTCAATGCGCAGGATGATGGGCAGCAGGC
CAGTGCCGAGATCCGTCGCACCCGTTTTGGCGTACCGCATATCCGGGCTCAGGATGAACGCGGGTTG
GGTTATGGCATCGGCTATGCCTACGCCCAGGACAACCTGTGCCTGCTGGCCAATGAGATCGTCACGG
TCAACGCCCAGCGCTCGCGCTATTTCGGGCCGCAGCAAGTCACGGTCGAACAGCGGGAGAACCGCGT
CAGCGATGTGTTTTTCAGTTGGCTCAACACACCGCAAGCGGTTTCCGGTTTCTGGCAGGCCCAGACGC
CTCAAGTGCAGCAACTGGTCGAAGGCTACGTGGCGGGCTACAACCGTGCGTTGGCCGAGCGCAAGG
CCAAAGGCCTGCCGGAACAATGTGCCGGCGAGTGGGTGCGGCCGATCACGGCGCTGGACCTGGTCA
AGTTGACTCGTCGACTGTTGGTGGAAGGCGGCGTCGGCCAGTTCGCCGAGGCCCTGGCTGGCGCGCA
GCCGCCCCAGGCGACGGCACTTGCGGGTGCTTCGGCCAACGGCTTCGCTGCCGCCGCAACCCGCCAG
CAGCGTTTTGCCCTGGAGCGTGGCAGCAATGCGCTGGCCATCGGCAGTGAGCGCTCGTTCAACGGCC
GTGGAATGTTGCTGGCGAATCCGCATTTTCCATGGTTGGGCGGCATGCGTTTCTACCAGATGCACTTG
ACCATTCCCGGCAAGCTGGATGTCATGGGAGCGGCCCTGCCGGGCCTGCCGATGATCAACATCGGCT
TCAGCCAGCACCTGGCCTGGACCCACACCGTCGACAGCTCAAAACACTTCACCCTGTACCGCCTGCA
ACTCGACCCGAAAGACCCGACCCGCTACCTGCTCGACGGCAAGTCTGTGCCGATGAGCCAACAGACG
GTCGCGGTAGACATCAAGCAACCCGACGGCCAAGTGCAGACGATTTCCCGAGTGGTCTATGGCTCGC
AGTTTGGCCCGATCGTGCAATGGCCCGGCCGGCTGGACTGGGATAACCGGTTCGCCTACAGCCTGCG
GGACGCGAACCTGGAAAACGATCGCGTGCTGGCCCAGTGGTACGCCATGAACAAGGCGGTCACGCT
CAAGGATCTGCAGGACGCCGTCCATGAGATCCAGGGCATTCCCTGGGTCAATACCCTGGCGGTGGAC
GATCAGGGGCAAAGCCTCTACATGAACGTGTCGGTGGTACCGAACGTCGACGCCGATAAGCTGGCCC
G

GTGCAGCGACCCCAGTGCCGGGCTGCAACTGATCGTGCTGGATGGGGCTCGCAGCGAATGTGCCTGG
GCTATTGATCCCAAGGCGGCGCAGAAAGGCATCTATGCCGCCGACAGGCTTCCGCAGTTGCTGCGCC
GCGATTATGTGCAGAACTCCAACGATTCGGCCTGGATGGTCAACCCGTCGCAGCCGCTGTCCGGTTA
TTCCCCATTGATCAGCCAGCAAGGCCAGCCGCTGGGGTTGCGGGCGCGGTTTGCGCTGGAGCGGATG
GCCGCGCTGGCCAAGGATGGGCCGGTGAAGGTGGAGGATTTGCAGCGCATGGTCATGGACGATCAG
GTTTACCTGGCCGACCAGGTGATGCCGGATCTCCTCGGCGTTTGTGCCGGTGACCTGGGGCCGGATG
CGTCGGCCCTGGTCGAGGTTTGTGCCAGCCTCAAGGCCTGGGATCGTACGGCGGGCCTGAAGAGCGG
CCTGGGGTTCGTGCATTTCCAGCACATCATGGACGAGGTGCGGGCTGCCCCGGGTTCGTGGCGCGTC
GCATTCGACCCCAAGGACCCGCAACACACCCCACGAGGCCTGGCGATCGAGCGACCGCCAGTGCTC
AAGGCCGTGCGCGAAGCCATGCTGGCCTCGGTGAAGGCGGTGAAGGCCGCCGGGTTGTCGAAGGAC
AGCCAATGGCAAGATGTCCAGGTGTCCAGCAGCGGCGGTCGACAAACGCCGATCCATGGCGGCCCC
GGGGAGCTGGGCATCTATAACGCGATCCAGAGCGTGCCGGGGGCGAACGGTAAGCGGGAG
GTGGTCAGCGGTACCAGCTATCTGCAAGTGGTGACGTTCGACGGTAAAGGTCCGCAGGCCCAGGGAT
TGTTGGCTTTTTCCATTTCCAGCGATCCGGCATCGCCTTACTCGGCAGATCAGACCCAGGCGTTTTCG
CAGAAGCAGTGGAGTGTGCTGCCATTCACCGAACAGCAGATCAAGGCCGATCCGCACTATCAGGCA
CTGATTATCCGTGAACGTGATGAGGCGGGCAGGGTGGCGACGCAGTAA

mupX Sequence:

ATGACCAGTGCATTTCACTTTCCAGAATACGACCTGTTGGACGGCATTGCGCTCGCGGATATGGTGC
GTCGCAAAGAAGTAACGCCTTCGGAGTTAGTGAGGGCGGCCGTTCAGCGAATCGACGGACGTAATG
GCCCACTCAACGCAGTCGTGCATCTGCTTGAAGATGAGGCAAAAAGCCAATGCCGGCTGCCGCTTGC
CGATGGCCCGTTGTCAGGGGTGCCCATACTGATCAAGGATCTGCTCGCGGAAATCGAGGGCTGCCCA
ACCCGTAATGGCTCGCGTCTATTCAAGCACTACGTTGCCCGGGAAGACTCGCAGACCATAAAACGCT
ATCGAAAGGCCGGGTTGATCTTTGTCGGAAAAACCGCCACGCCTGAGCTGGGGTTGCATCCCTATAC
CGAATCCGATGCAACCGGCATTACACGCAACCCCTGGAACCTCGGTTTGTCACCGGGAGGGTCCAGC
GGCGGCGCTTGTGCAGCGGTGGCGGCGGGGATGACGCCCATAGCCCATGGCAGCGATGGGGGCGGG
TCAATTCGATTGCCTGCTTCCCATTGCGGCGTTTTCGGCCTTAAGCCAACCCGTGGCCGGTCTCCCAG
TGGCCCGCATTTTTCAGAGCTATGGCAAGGACTGGTGGTTGAGCATGCAGTCTCCAGAAGCGTTCGT
GACAGTGCGGCCATGCTCGATATTCTGATTGCGGGAAGTGATGACGCCGACGCGTACCGGTGGCCGG
CGCCTGAAGAGTCTTTTCTATCTTCGACCTGCAACACGCCTGGCCGATTGCGCATCGCTTACACCTTC
CAGCCTTTTCTAGGCGGGGAACTGAGTCCCGATTGTCGAGCGGCAGTAGAGAGCAGTTTGCAACTGT
TGGCGGACCTTGGGCACGATGTTGTCGAGGCTCATCCGCCCTTGGCGTCCGCTGACCAATTGTGTGA
GGCGATGCTGACCATCGTGTGCGGGGAAATGGCAAGTCTGGTGGAGAACGCGGGGCGGATGCTCGA
TCGCGTGGCGACTTATGAAGATTTCGAGCCTGGCACTTGGGCACTGGCTCGGCAAGGCCATATTTTC
AAAGCGGTCCACCTGGCCAGAATGA GAGAGTTGGCGCTTCACCAGGGGCGCATA
ATGAGTGCGTTCCATGAAGACTATGATGTGCTGGTGACCCCAGTGGTCAATCAACTTCCATCGGCCG
TGGGCGCTTTTCGCTTGAGTCCTTTGGAGGCGCGCCTGTCGCGTAAAGTATTGGGTCAATGGGGGCA
GGACTGGCCGCTGCGCTTGGGCAACCGGTTACCCCAAACATCTCGTCAGGTCATGGAGTACATGGGC
TGGTCGACACCCTTTAATATGAGCGGGCAGCCCGCGATGAGTGTGCCGCTGTTCTGGAACGAGGCAG
GTCTTCCGATCGGTACGCAGTTCGTTGCAAAGATGGGTAACGAAGCAATGTTACTGCAACTTGCGCG
GTCGCTTGAAATGGCACGCCCGTGGCAACATCGACGCCCGCCTCAGGGCCAGCGGGCGTCACTTGGG
TAG
